# Supplementary material for: The Systems Biology Research Tool: evolvable open-source software
Source: BMC Syst Biol. 2008 Jun 29;2:55. doi: 10.1186/1752-0509-2-55 (PMC2446383; doi:10.1186/1752-0509-2-55)
Supplement: Additional file 1 — SBRT Archive. An archive of the current version of the Systems Biology Research Tool. [file 1752-0509-2-55-S1.zip › sbrt-1.4.0/doc/developers_guide/api/sbrt/kernel/math/class-use/Polynomial.Term.html]

Uses of Interface sbrt.kernel.math.Polynomial.Term


|  |  |  |
| --- | --- | --- |
| **Overview** \| **Package** \| **Class** \| **Use** \| **Tree** \| **Deprecated** \| **Index** \| **Help** | |  |
| PREV   NEXT | **FRAMES**    **NO FRAMES**     **All Classes** |  |


---


## **Uses of Interface sbrt.kernel.math.Polynomial.Term**

| Packages that use Polynomial.Term | |
| --- | --- |
| **sbrt.kernel.math** | Provides classes and interfaces for creating objects relevant to mathematics. |

Uses of Polynomial.Term in sbrt.kernel.math

| Subinterfaces of Polynomial.Term in sbrt.kernel.math | |
| --- | --- |
| `static interface` | `LinearComb.Term<V>`             This interface is used to represent the terms of linear combinations. |
| `static interface` | `QuadraticExpr.Term<V>`             This interface is used to represent the terms of quadratic expressions. |

| Classes in sbrt.kernel.math that implement Polynomial.Term | |
| --- | --- |
| `static class` | `AbstractLinearComb.Term<V>`             This class is a skeleton implementation of `LinearComb.Term`. |
| `static class` | `HashLinearComb.Term<V>`             This class is a concrete implementation of `LinearComb.Term`. |

| Methods in sbrt.kernel.math that return Polynomial.Term | |
| --- | --- |
| `Polynomial.Term<V>[]` | `Polynomial.getTermArray()`             Returns an array containing all of the terms of this polynomial. |

---


|  |  |  |
| --- | --- | --- |
| **Overview** \| **Package** \| **Class** \| **Use** \| **Tree** \| **Deprecated** \| **Index** \| **Help** | |  |
| PREV   NEXT | **FRAMES**    **NO FRAMES**     **All Classes** |  |


---
